# Toxic and heavy metals contamination assessment in soil and water to evaluate human health risk

**DOI:** 10.1038/s41598-021-94616-4

**Published:** 2021-08-20

**Authors:** Waqar Ahmad, Rima D. Alharthy, Muhammad Zubair, Mahmood Ahmed, Abdul Hameed, Sajjad Rafique

**Affiliations:** 1 Department of Chemistry, University of Gujrat, Gujrat, Pakistan; 2 Department of Chemistry, Science and Arts College, Rabigh Campus, King Abdulaziz University, Jeddah, 21577 Saudi Arabia; 3 Renacon Pharma Limited, Lahore, 54600 Pakistan; 4 Department of Chemistry, University of Sahiwal, Sahiwal, Pakistan

**Keywords:** Environmental sciences, Chemistry

## Abstract

Due to urbanization and industrialization, there has been an increase in solid waste generation and has become a global concern and leakage of leachate from landfills contaminate the soil and groundwater and hence can have a severe impact on human health. The present study aimed to determine the composition of toxic metals (Cr, Mn, Cu, As) and heavy metals (Cd, Ba, Hg, Pb) in soil and water by an inductively coupled plasma optical emission spectrometer (ICP-OES). To ensure accuracy during the analysis of Cr, Mn, Cu, As, Cd, Ba, Hg, and Pb in real samples, certified reference material (CRM, SRM 2709a) of San Joaquin soil and water (SRM 1640a) were analyzed and results were presented in terms of % recovery studies. The mean concentration of all the metals in soil and water did not exceed the limit set by the European Community (EU), WHO, and US EPA except Cu where the permissible limit defined by the EU is 50–140 mg/kg in soil. The soil is uncontaminated to moderately contaminated with respect to all metals except the Cu and Pb. Among the average daily dose (ADD) of soil, ADD_*ing*_ and ADD_*inh*_ for children had the maximum dose for all metals than adults while ADD_*derm*_ was higher in adults. Hazard quotient (HQ) trend in both adults and children was found in order HQ_*ing*_ > HQ_*derm*_ > HQ_*inh*_ of soil for all metals except Ba which followed HQ_*ing*_ > HQ_*inh*_ > HQ_*derm*_. Hazard index (HI) values of soil for Cr and Pb in children were 7 and 7.5 times higher than adults respectively. Lifetime cancer risk (LCR) value for Cr by different exposure pathways of soil was 5.361 × 10^−4^ for children which are at the lower borderline of risk for cancer.

## Introduction

Despite much awareness about solid waste, it is increasing day by day due to anthropogenic activities throughout the world. Due to urbanization and industrialization, there has been an increase in solid waste generation and has become a global concern^1^. Effective management of solid waste is being geared up in both developed and developing countries due to its adverse effect and impact on human health and the environment respectively^2^. Among the waste management methods, landfilling is a primary method of disposing of and has gained acceptance because it is simple and has considerable advantages like economic efficiency and low technological barriers^3^. If the landfill site does not have an appropriate system like leachate liner and its collection then it possesses a potential risk to soil and groundwater aquifer near the area surrounding the landfill site. The leakage of leachate from landfills contaminates the groundwater, soil, surface water, and natural ecosystems especially when the leachate is released uncontrolled, and hence can have a severe impact on environmental and human health^4,5^. Moisture in soil under favorable environmental factors generates the leachate from landfills which can enter into the soil and aquatic environments^6,7^.

The presence of several pollutants including suspended particles (organic and inorganic), toxic (TMs), and heavy metals (HMs) in landfill leachate is a matter of concern and it can pose a serious threat to public health as well as ecotoxicological impacts on terrestrial and aquatic ecosystems^1,8,9^. Soil is an important part of the terrestrial ecosystem and it is the ultimate sink for HMs and becomes a medium to spread them into water bodies, organisms, and atmosphere. HMs are persistent and accumulative in soil and increase the toxicity of soils after combining with inorganic and organic matters. So HMs can accumulate in the food chain, then enter the human body through food consumption. HMs can also accumulate in the human body via dermal contact absorption and direct ingestion and inhalation^10,11^. Drinking of water and inhalation of soil particles have been identified as the major pathway for human exposure to toxic metals^12,13^.

The impact of Cr upon human health is dependent upon the oxidation state because Cr (III) is an important ingredient of human diet and plays a significant role in human metabolism whereas Cr (VI) is highly carcinogenic as well causing cardiovascular and liver diseases^14^. Mn plays an important role in the metabolism of carbohydrates, cholesterol, and amino acids metabolism. Higher concentrations of Mn can cause several effects like male infertility, neurological disorders, birth disability, and bone defects^15^. Cu has a significant role in the physiological function of the human body but excessive ingestion can have unfavorable effects on human health. The Cu in excess amount has noxious effects on the intestine, liver and damage the stomach^16–18^. As if ingested in higher concentrations can lead to skin cancer, dermal lesions, angiosarcoma, peripheral neuropathy, and vascular disease^19,20^. Cd if present in excess can damage the lungs, liver, induces osteotoxicity and nephrotoxicity, pulmonary adenocarcinomas, prostatic proliferative lesions, pulmonary adenocarcinomas and disturbs the immune system of the body^21,22^. Ingestion of Ba can cause changes in heart rhythm or paralysis in humans and may cause mortality if did not seek medical advice. Exposure to Ba for a short period may experience difficulties in breathing, irregulation in blood pressure, diarrhea, vomiting, diarrhea, and muscle weakness^23^. Cysteine residue of protein and Hg (II) form a covalent bond that results in depletion of cellular antioxidants, so Hg produces reactive oxygen reactive species (ROS) as a result of oxidative damage and oxidative damage describe the molecular mechanism of toxicity. The carcinogenic process initiates as a result of DNA damage in cells which is caused by ROS because ROS play a major role in the metal induced cellular responses^24,25^. Excessive concentration of Pb in the blood can cause hypertension, damage the skeletal, immune system, endocrine, reduces intelligence potential in kids and among adults it also affects the functioning of kidney and heart^26–28^.

So the presence of TMs (Cr, Mn, Cu, and As) and HMs (Cd, Ba, Hg, Pb) in soil and water can cause serious damage to human health. Contamination of soil and groundwater with TMs and HMs surrounding the industrial landfills becomes one of the most challenging environmental and health issues because of their persistence, toxicity, bioaccumulation, and non-biodegradability^29–31^.

Within this context, our study is to investigate the TMs and HMs composition in the soil and groundwater by inductively coupled plasma-optical emission spectroscopy (ICP-OES). Therefore, the aim of this study to assess the contamination level of the aforementioned metals in the soil and groundwater near the industrial landfill sites in Sialkot (an industrial city in the province of Punjab-Pakistan). Geo-accumulation index (I_geo_), contamination factor (CF) and potential ecological risk index (PERI) were determined to assess pollution, evaluate the pattern of contamination and determine the potential risk due to exposure to ecological sensitivity, concentration and toxicity of TMs and HMs in soil. Information about these metals is equally important for assessing their potential risk to human health, so the average daily dose (ADD), the non-carcinogenic target hazard quotient (THQ), and carcinogenic risk (CR) coefficients were demonstrated to evaluate the human health risk.

To the best of our knowledge, no previous study was conducted for the estimation of TMs and HMs in soil and water near the industrial landfill site. So different regions surrounds the landfill site were chosen to study the TMs and HMs composition in soil and water to estimate the potential health risk of these metals on human health and ecosystems. This study also aimed to provide helpful information by computing various contamination factors for scientific management of industrial activities for pollution control in relation to ecosystems and human health risk.

## Materials and methods

### Study area

**Figure 1:**
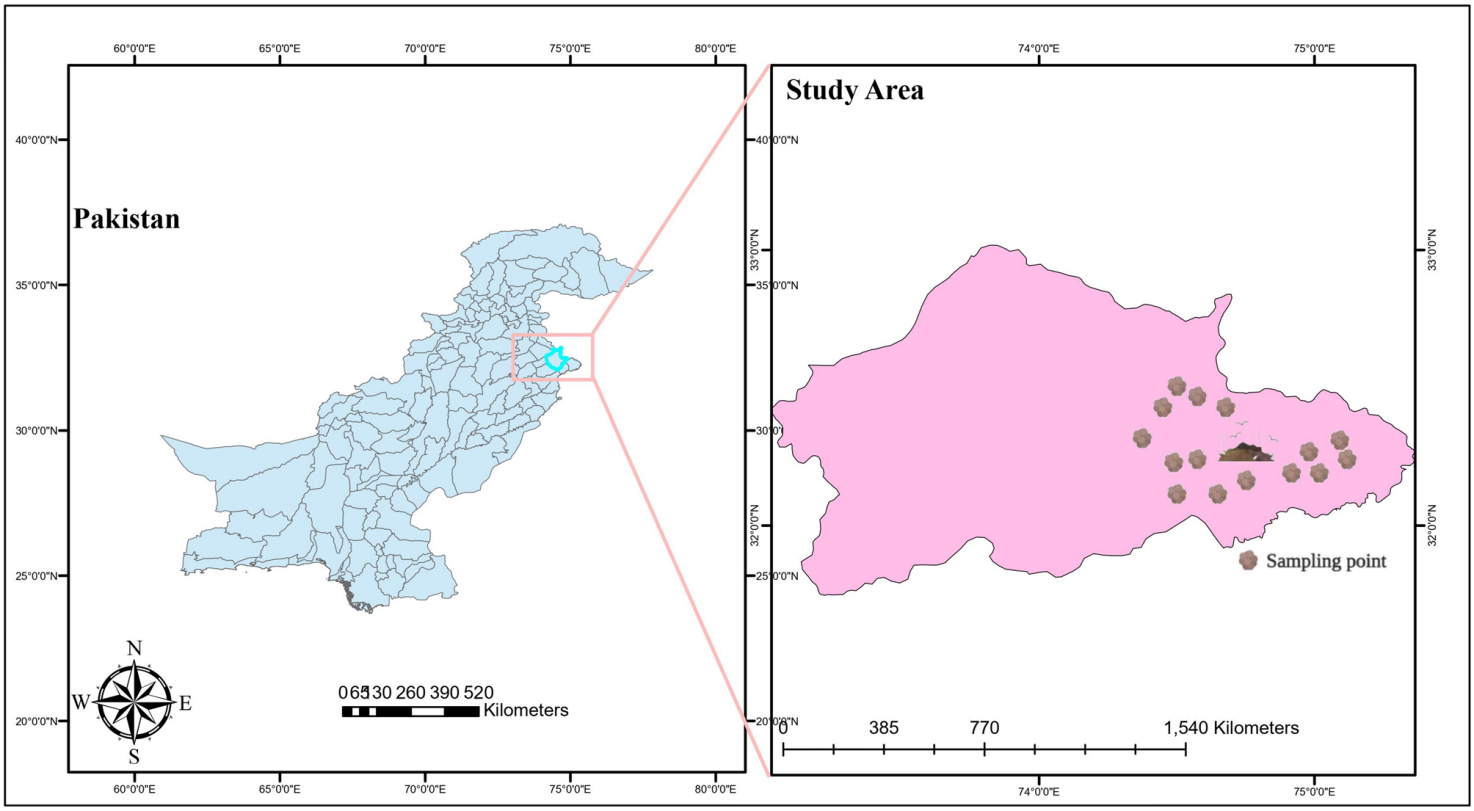
Study area.

The study was conducted in the industrial city of Sialkot (32° 29′ 33.65′′ N and 74° 31′ 52.82′′ E) in the province of Punjab-Pakistan (Fig. 1). The city is renowned for manufacturing of leather items including sports and surgical instrument. The study area is based on 19 km^2^. The total population of the city was 477,396 in 2019. The mean annual temperature of the study area was 29.6 °C, June is the hottest month with a temperature of 39.0 °C and the coldest month is January with a temperature of 6.0 °C. The average temperature range in April 19–34 °C and in October 18–31 °C, with average humidity about 50–69% respectively. Roughly 3/4 of the waste produced from leather industries, while the remaining is contributed by surgical equipment manufacturing industries. Solid waste from industries dump in empty plots/yards and these landfills were not lined but had compacted clay at the base to minimize seepage of leachate into the subsurface environment.

### Reagents and solutions

Hydrofluoric acid (HF, 48% m/m), Nitric acid, (HNO_3_, 65% m/m), for the preparation of solution for digestion of soil, were purchased from Hajvery Scientific Store, Lahore-Pakistan originate to Merck, Germany. Certified reference material of each element (1000 mg L^−1^, Merck, Germany) was employed for instrument calibration after diluting it with HNO_3_ (2% v/v). Ultrapure water (18 MΩ.cm resistivity) for the dilution of all solutions was prepared by the GenPure water system (Thermo Scientific, USA). All glassware and materials used in the procedure were soaked in nitric acid (20% v/v) and then exhaustively washed with ultrapure water. Certified reference materials (SRM 2709a) of San Joaquin soil and water (SRM 1640a) were used as a reference.

### Collection, preparation, analysis of soil and water samples

For soil analysis, a systematic sampling approach was adopted for the collection of soil samples by distributing the study area into four regions such as east (region I), west (region II), north (region III), south (region IV). From each region, five samples were collected at a distance of 10–50 m (after a consecutive distance of 10 m) from the landfill site and in total twenty samples were collected (5 samples × 4 regions). About 500 g sample was collected from each sample point a depth of 0.5–20, 20–40, 40–60 cm while the top 0.5 cm of surface soil was removed before sampling. Each sample is comprised of three subsamples which are pooled and homogenized to form a representative sample. The representative samples were coded as SLNS1-SLNS5, SLSS1-SLSS5, SLES1-SLES5, and SLWS1-SLWS5 mean five samples from each region and transported to the laboratory after packed in a polyethylene bag. The samples were air-dried at 35 °C for 72 h using an air circulation oven (Vision Scientific, Korea), crushed, passed through a sieve of mesh size No. 10, and stored in polyethylene bottles before acid digestion followed by metal analysis. 1 g of soil representative sample was weighed and transferred to porcelain crucible already contained 10 mL mixture of HNO_3_ and HF (1:1), the residue was formed after drying in a water bath after which the residue was dissolved in 20 mL HNO_3_ (2 M). Whatman No. 41 filter paper was used to remove the particles in the resulting solution, then the solution was transferred to a 100 mL measuring flask and made the volume up to mark with ultrapure water^32^.

For water analysis, twenty samples were also collected from boreholes in 1 km^2^ surrounding of landfill site were coded as SLNW1-SLNW5, SLSW1-SLSW5, SLEW1-SLEW5, and SLWW1-SLWW5, to eliminate immobile water samples were fetched after pumping for 15 min while the samples were collected into clean polyethylene bottles having a capacity of 1.0 L. To minimize the adsorption and precipitation on the walls of bottles, the samples were acidified to a pH < 2.0 with HNO_3_^33^. Cr, Mn, Cu, As, Cd, Ba, Hg, and Pb in soil and water samples were determined by ICP-OES (Thermo Fisher Scientific, iCAP 7400), operating conditions are shown in Table 1 and all measurements were made in triplicate while Thermo iteva software was used to control the system.

**Table 1:** ICP-OES operational parameters.

ICP-OES	Thermo Fisher Scientific, iCAP 7400 with the radial viewed plasma equipped with echelle type 52.91 grooves/mm ruled grating
RF power	1250 W
Radial viewing height	8 mm
Plasma coolant gas flow rate	15.0 L min^−1^
Auxiliary gas flow rate	1.0 L min^−1^
Nebulizer gas flow	0.65 L min^−1^
Nebulizer type	V-groove, pressure 240 kPa
Sample uptake rate	1.5 mL min^−1^
Pump rate	35 rpm during flushing, 20 rpm during the analysis
Spectral lines (nm)	Cr: 267.716, Mn: 257.610 Cu: 327.393 As: 188.980, Cd: 214.439, Ba: 455.403 Hg: 194.164 Pb: 220.353

### Analytical figures of merit

Accuracy of acid digestion method for soil analysis was assessed by analysis of CRM and recovery (%) was calculated while the precision was evaluated by the repetition of acid digestion procedure in replicates of ten (n = 10) under the same conditions and results of repeatability are expressed in % RSD. Detection limits including LOD and LOQ were estimated as LOD = 3 σ/S, LOQ = 10 σ/S respectively where σ is SD (standard deviation) of analytical blank measurement (n = 18) while S is the slope of the calibration curve (y = mx + b)^34,35^.

### Toxic and heavy metals contamination assessment in soil

Geo-accumulation index (I_geo_), contamination factor (CF) and potential ecological risk index (PERI) can be used to assess pollution, evaluate the pattern of contamination and determine the potential risk due to exposure to ecological sensitivity, concentration and toxicity of TMs and HMs in soil. I_geo_ was calculated by using formula^11^.

a $${\text{I}}_{{{\text{geo}}}} = \log_{2} \left( {\frac{{{\text{C}}_{{\text{n}}} }}{{1.5{\text{B}}_{{\text{n}}} }}} \right)$$

where C_n_ is the concentration of an element in the soil sample while B_n_ is the geochemical background value of non-effected soil at the site of city. The constant 1.5 allowed us to minimize effect variation in background concentration due to lithogenic impacts^36^. Soil quality can be distinguished by I_geo_ index classification as follows, I_geo_ ≤ 0 (uncontaminated), 0 < I_geo_ < 1 (uncontaminated to moderately contaminated), 1 < I_geo_ < 2 (moderately contaminated), 2 < I_geo_ < 3 (moderately to heavily contaminated), 3 < I_geo_ < 4 (heavily contaminated), 4 < I_geo_ < 5 (heavily to extremely contaminated), 5 < I_geo_ (extremely contaminated). CF was estimated by following equation^11^

b $${\text{CF}} = \frac{{{\text{C}}_{{\text{n}}} }}{{{\text{C}}_{{\text{b}}} }}$$

where C_n_ is the concentration of an element in the soil sample while C_b_ is the geochemical background value of non-effected soil at the site of city. Soil quality can be classified by CF value as follows, CF < 1 (low contamination), 1 ≤ CF < 3 (moderate contamination), 3 ≤ CF < 6 (considerable contamination), and CF ≥ 6 (very high contamination)^11^.

The potential ecological risk of individual metal element can be determined as follows^37^

c $${\text{E}}_{{\text{r}}}^{{\text{i}}} = {\text{C}}_{{\text{r}}}^{{\text{i}}} \times {\text{T}}_{{\text{r}}}^{{\text{i}}} = \left( {\frac{{{\text{C}}_{{\text{S}}}^{{\text{i}}} }}{{{\text{C}}_{{\text{n}}}^{{\text{i}}} }}} \right) \times {\text{T}}_{{\text{r}}}^{{\text{i}}}$$

where

- ${\text{C}}_{{\text{S}}}^{{\text{i}}}$ = concentration of an element in the soil sample
- ${\text{C}}_{{\text{n}}}^{{\text{i}}}$ = geochemical background value of non-effected soil
- ${\text{T}}_{{\text{r}}}^{{\text{i}}}$ = toxic response factor for each metal

Toxic response factor was taken 2 for Cr, 5 for Cu and Pb, 10 for As, 40 for Hg and 50 for Cd^38^. Soil quality can be classified by E_r_ value as follows, E_r_ < 40 (low risk), 40 ≤ E_r_ < 80 (moderate risk), 80 ≤ E_r_ < 160 (considerable risk), 160 ≤ E_r_ < 320 (high risk), and E_r_ ≥ 320 (very high risk).

### Human health risk assessment

To evaluate the human exposure to TMs and HMs in soil and water, an estimation of risk was determined by using average daily dose (ADD), the non-carcinogenic target hazard quotient (THQ), hazard index (HI), and lifetime carcinogenic risk (LCR) coefficients^9,11,39^.

#### Average daily dose

ADD of three exposure pathways of soil including ingestion (ADD_*ing*_), inhalation (ADD_*inh*_), and dermal contact (ADD_*derm*_) in mg/kg/day of metals including Cr, Mn, Cu, As, Cd, Ba, Hg, and Pb was calculated by using the formula^11,37,40,41^.

d $${\text{ADD}}_{ing} = {\text{C}} \times \frac{{{\text{IngR}} \times {\text{EF}} \times {\text{ED}}}}{{{\text{BW}} \times {\text{AT}}}} \times 10^{ - 6}$$

e $${\text{ADD}}_{inh} = {\text{C}} \times \frac{{{\text{InhR}} \times {\text{EF}} \times {\text{ED}}}}{{{\text{PEF}} \times {\text{BW}} \times {\text{AT}}}}$$

f $${\text{ADD}}_{derm} = {\text{C}} \times \frac{{{\text{SA}} \times {\text{AF}} \times {\text{ABF}} \times {\text{EF}} \times {\text{ED}}}}{{{\text{BW}} \times {\text{AT}}}} \times 10^{ - 6}$$

where C is the concentration of TMs and HMs in mg/kg, IngR is ingestion rate in mg/day (IngR = 200 for children and 100 for adults), EF exposure frequency in days/year (180), ED is exposure duration (6 years for children and 24 years for adults), BW is the average body weight (child = 15 kg and adult = 70 kg), AT is the average time (365 × ED), InhR is inhalation rate in mg/cm^2^ (20 for both adult and children), PEF is particle emission factor in m^3^ kg^−1^ (1.36 × 10^9^ for both adult and children), SA is the surface area of the exposed skin in cm^2^ (2145 for adult and 1150 for children), AF is the skin adherence factor for the soil in mg cm^2–1^ (0.2 for adult and 0.07 for children), ABF presents the dermal absorption factor (0.03 for As and 0.001 for other metals). While ADD_*ing*_ and ADD_*derm*_ of water in mg^−1^ kg^−1^ day^−1^ of metals including Cr, Mn, Cu, As, Cd, Ba, Hg, and Pb was calculated by using the formula given above for soil whereas IngR is ingestion rate of water is 2.2 L day^−1^ for adult and 1.5 L day^−1^ for children, EF is 365 days year^−1^, SA is 5700 cm^2^ and rest of parameters are same as mentioned above.

#### Total hazard quotient and index

THQ is the ratio of ADD (from three exposure pathways) and RfD (chronic reference dose for each metal in mg/kg BW/day) which is typically used to estimate the potential non-carcinogenic risk of metals exposure to humans in three different pathways^36^.

g $${\text{THQ}} = \frac{{{\text{ADD}}\left( {{\text{ingestion}},\;{\text{inhalation}}\;{\text{or}}\;{\text{dermal}}} \right)}}{{{\text{RfD}}}}$$

RfD in mg/kg BW/day for Cr, Mn, Cu, As, Cd, Ba, Hg, and Pb respectively are presented in Table 2 whereas THQ < 1 considers the exposed population experience no significant health risk.

**Table 2:** RfD and CSF values used in this study^39,42^.

Metal	RfD_*ing*_	RfD_*inh*_	RfD_*derm*_	CSF_*ing*_	CSF_*inh*_	CSF_*derm*_
Cr	3 × 10^−3^	2.86 × 10^−5^	6 × 10^−5^	0.5	–	41.0
Mn	140 × 10^−3^	140 × 10^−3^	1.8 × 10^−3^	–	–	–
Cu	4 × 10^−2^	4.02 × 10^−2^	1.2 × 10^−2^	–	–	–
As	3 × 10^−4^	3 × 10^−4^	1.23 × 10^−4^	1.5	1.5	1.5
Cd	1 × 10^−4^	1 × 10^−4^	1 × 10^−5^	0.38	–	6.3
Ba	7 × 10^−2^	1.43 × 10^−4^	4.9 × 10^−3^	–	–	–
Pb	3.5 × 10^−3^	3.25 × 10^−3^	5.25 × 10^−4^	0.0085	–	0.042
Hg	3 × 10^−4^	3 × 10^−4^	2.1 × 10^−5^	–	–	–

Whereas the hazard index (HI) is equal to the sum of all expected HQs (non-carcinogenic risks) through inhalation, oral, and dermal, pathways and is employed to compute the total potential non-carcinogenic risks of different contaminants through the 3 exposure routes mentioned above^11^.

h $${\text{HI}} = \Sigma {\text{HQ}} = {\text{HQ}}_{ing} + {\text{HQ}}_{inh} + {\text{HQ}}_{derm}$$

If the value of HI ≤ 1 then it indicates no significant risk of non-carcinogenic effects. However, when HI > 1, there is a probability of non-carcinogenic effects occurring, and the probability increases with a rising value of HI^43,44^.

#### Carcinogenic risk assessment

Cancer risk for lifetime exposure (LCR) of Cr, As, Cd, Pb, and Hg were estimated to determine the health risk by calculating the cumulative life cancer risk rating using the formula below for each exposure pathway^45^:

i $$\begin{aligned} {\text{LCR}} & = {\text{ADD}}\left( {{\text{ingestion}},\;{\text{inhalation}}\;{\text{or}}\;{\text{dermal}}} \right) \times {\text{CSF}} \\ & = \Sigma \,{\text{cancer}}\;{\text{risk}} = {\text{cancer}}\;{\text{risk}}_{ing} + {\text{cancer}}\;{\text{risk}}_{inh} + {\text{cancer}}\;{\text{risk}}_{derm} \\ \end{aligned}$$

where CSF is the cancer slope factor which is given in Table 2 for each metal for three exposure pathways. Whereas LCR < 10^−6^, LCR > 1 × 10^−4^, and LCR 1 × 10^−6^ to 1 × 10^−4^ indicates no carcinogenic risk, high risk of developing cancer, and signifies acceptable risk to humans respectively.

## Results and discussion

### Analytical figure of merit

Accuracy is a very important and prime factor in analytical results because these results are subject to errors which cause the results to differ from the true concentration of determinants. The acquired results affecting the ability of decisions based on these results. Various factors such as purity of reagents, standards, the magnitude of matrices effects, instrument's stability, and environmental condition of the laboratory. So to ensure accuracy during the analysis of Cr, Mn, Cu, As, Cd, Ba, Hg, and Pb in real samples, CRM (SRM 2709a) of San Joaquin soil and water (SRM 1640a) were analyzed and results were presented in terms of % recovery studies (Tables 1S and 3). The recovery results were obtained in ranged between 93.2 and 107.6% that determines the excellent extraction efficiency.

**Table 3:** Measured and certified values of the metals in SRM 2709a and SRM 1640a.

Metal	Certified value SRM 2709a (µg g^−1^)	Measured value $\left( {\overline{x} \pm ts/\surd n} \right)$	Certified value SRM 1640a (µg kg^−1^)	Measured value $\left( {\overline{x} \pm ts/\surd n} \right)$	% Recovery SRM 2709a/SRM 1640a
**Toxic metals**					
Cr	130 ± 9	132.9 ± 2.7	40.22 ± 0.28	39.20 ± 1.52	102.2/97.5
Mn	529 ± 18	527.9 ± 1.8	40.07 ± 0.35	39.13 ± 1.32	99.8/97.6
Cu	33.9 ± 0.5	33.5 ± 0.67	85.07 ± 0.48	85.34 ± 0.58	98.8/100.3
As	10.5 ± 0.3	11.3 ± 0.52	8.010 ± 0.067	8.00 ± 0.59	107.6/99.8
**Heavy metals**					
Cd	0.371 ± 0.002	0.362 ± 0.04	3.961 ± 0.072	3.690 ± 0.279	97.6/93.2
Ba	979 ± 28	973.3 ± 7.8	150.60 ± 0.074	151.88 ± 1.224	99.4/100.8
Hg	0.09 ± 0.02	0.09 ± 0.004	–	–	100/–
Pb	17.3 ± 0.10	17.8 ± 0.29	12.005 ± 0.040	11.887 ± 0.389	102.9/99.0

($\overline{x} \pm ts/\surd n$) = mean ± CI (*p* < 0.05, n = 6), CI = Confidence interval.

A linear dynamic range of 0.2–1.0 μg mL^−1^ for Mn, and Cu, 0.01–500 μg L^−1^ for Cr, As, Cd, and Ba, while 0.02–1000 μg L^−1^ for Pb and Hg was selected and linear calibration curve in the form of y = mx + b was obtained by plotting the peak height (y) of each concentration in triplicate, against the nominal concentration. Where “m” represented the slope of the calibration curve and “b” indicated the intercept. The linear regression equation was demonstrated (Table 2S) and tabulated the necessary parameters. LOD and LOQ determined by proposed methods are as shown in Table 2S. The lower values of detection limits indicate that the method provided adequate sensitivity. The precision study in terms of repeatability (n = 10, Table 3S) was performed and % RSD was calculated. The % RSD values ranged from 0.20 to 6.40 while 0.42 to 4.06 (Table 2S) were obtained during the analysis of CRM (SRM 2709a) of San Joaquin soil and water (SRM 1640a) respectively.

### Application to real samples

The validated acid extraction procedure was applied to soil samples for extraction of Cr, Mn, Cu, As, Cd, Ba, Hg, and Pb, while the water samples were only acidified with to a pH < 2.0 with HNO_3_ subsequently theses were determined by ICP-OES, and results are presented in Table 4.

**Table 4:** Toxic and heavy metals concentration (mg kg^−1^) in soil and water of Sialkot city (n = 20).

Region	Concentration (mg/kg)	Cr (soil/water)	Mn (soil/water)	Cu (soil/water)	As (soil/water)	Cd (soil/water)	Ba (soil/water)	Hg (soil/water)	Pb (soil/water)
I	Min	114.0/.001	270.0/0.020	40.0/0.020	1.0/0.008	0.6/0.001	105.0/0.140	0.020/0.007	17.0/0.020
	Max	165.0/.002	320.0/0.025	80.0/0.030	2.1/0.011	1.0/0.001	125.0/0.160	0.025/0.010	35.0/0.040
	Mean	135.8/.001	291.6/0.021	61.2/0.026	1.6/0.010	0.8/0.001	117.0/0.148	0.022/0.008	23.2/0.033
II	Min	70.0/0.001	260.0/0.008	90.0/0.025	1.5/0.010	0.7/0.001	90.0/0.120	0.025/0.005	20.0/0.030
	Max	125.0/0.001	345.0/0.020	125.0/0.030	1.6/0.013	1.0/0.002	110.0/0.170	0.045/0.007	55.0/0.030
	Mean	95.2/0.001	300.2/0.017	111.0/0.026	1.5/0.011	0.8/0.002	100.2/0.139	0.038/0.006	33.4/0.030
III	Min	65.0/0.001	365.0/0.001	70.0/0.030	1.6/0.009	0.1/0.001	95.0/0.158	0.025/0.004	40.0/0.030
	Max	110.0/0.002	350.0/0.010	85.0/0.035	2.0/0.011	0.6/0.002	150.0/0.160	0.035/0.008	55.0/0.045
	Mean	93.5/0.001	302.6/0.006	80.0/0.032	1.9/0.010	0.4/0.002	113.9/0.160	0.029/0.006	49.6/0.037
IV	Min	145.0/0.001	290.0/0.030	30.0/0.040	0.5/0.009	0.6/0.001	93.0/0.160	0.005/0.008	18.0/0.030
	Max	535.0/0.002	410.0/0.040	60.0/0.060	0.9/0.013	0.9/0.002	110.0/0.200	0.011/0.010	20.0/0.045
	Mean	307.0/0.001	343.0/0.034	43.0/0.047	0.8/0.011	0.8/0.001	101.4/0.179	0.008/0.009	19.0/0.038
Total	Min	65.0/0.001	260.0/0.001	30.0/0.020	0.5/0.008	0.1/0.001	90.0/0.120	0.005/0.004	17.0/0.020
	Max	535.0/0.002	410.0/0.040	125.0/0.060	2.1/0.013	1.0/0.002	150.0/0.200	0.045/0.010	55.0/0.045
	Mean	157.87/0.001	309.36/0.019	73.80/0.033	1.44/0.010	0.7/0.002	108.13/0.156	0.024/0.006	31.30/0.034

Five samples each of soil and water from each region.

The metals were classified into two groups such as TMs and HMs and their concentration in soil was found in the following sequence Mn > Cr > Cu > As and Ba > Pb > Cd > Hg respectively whereas their concentration in water was found in the following order Cu > Mn > As > Cr and Ba > Pb > Hg > Cd respectively. The concentration of both TMs and HMs (Table 4S, supplementary data) decreased as sampling point distance increased from the landfill site so it was assumed that the point near the landfill site is most contaminated.

The mean concentration of Cr in water samples was found under the standard limit while its concentration in the soil samples exceeding the limit set by regulatory agencies like EU (soil: 100–150 mg kg^−1^)^46^, US EPA (water: 0.1 mg L^−1^), and WHO (0.05 mg L^−1^). Cr being strongly associated with soil and in this study area, its primary source is the migration of landfill leachate into surrounding soil where most of the landfill is waste of industries like tannery and chemical dying. Biological activities of soil are considerably affected by Cr contamination, chernozem's biota is highly affected by toxic effects of Cr and reduce its catalysis activity.

Cu and Mn concentrations among the TMs were found higher in both soil and water samples around the landfill sites because both the metals are used in the manufacturing of various items including wires, steel equipments, and alloys that find their way into the landfill. Their high concentration could be an indication of the migration of leachate rich in Cu and Mn into the surrounding soils. The concentration of Mn in soil was found within the reference range recommended by WHO (FAO/WHO: 20-10E04 mg kg^−1^) whereas Cu concentration was found higher than the permissible limit defined by the EU (50–140 mg kg^−1^)^46^ and FAO/WHO (36–75 mg kg^−1^)^47^. For water samples, the concentration of both the metals was found within the reference range recommended by WHO (Mn: 0.2 mg L^−1^ and Cu: 2.0 mg L^−1^)^48,49^. Cu is although an essential nutrient but its excess amount in the soil becomes toxic to some beneficial microorganisms and plants, it can inhibit the mineralization of phosphorus and nitrogen, also it has serious human health effects as well as toxic effects on fish and other aquatic organisms^50^. The concentration of Cu in the soil more than 300 mg kg^−1^ decreases the yield of rice, affects the germination of wheat, and reduces the biomass in shoots and roots of cabbage^51^. The mean concentration of As in both soil and water samples did not exceed the limit recommended by the European Community (soil: 20 mg kg^−1^) and WHO (water: 0.01 mg kg^−1^)^52^. There was a linear relationship observed between the concentration of As in soil and its transfer into the plants, and cultivation in As contaminated soil and irrigation with water severely affect the growth and results in less yield^46^.

The mean concentration of Cd in both soil and water samples was found under the standard limit set by regulatory agencies like EU (soil: 1–3 mg kg^−1^)^46^, US EPA (soil: 1.4 mg kg^−1^, water: 0.005 mg L^−1^)^53^ and WHO (0.005 mg L^−1^) but this soil is not suitable for gardening and agriculture because Cd concentration exceeds the limit set by US EPA (0.48 mg kg^−1^) and WHO (0.003 mg kg^−1^) respectively^54^. Cd being strongly associated with soil and in this study area, its primary source is the migration of landfill leachate into surrounding soil where most of the landfill is waste of industries where it is present as impurities in plant pigments, alloys, galvanized pipes, metal fittings, and solders. The high concentration of Cd has toxic effects on the beneficial microbes, disturb their metabolic process, and inhibit their growth. Cd has a lengthy biological half (10–35 years) in humans^55,56^. Ba and Pb concentrations among the HMs were found higher in both soil and water samples around the landfill sites because both the metals are used in the manufacturing of various items including lead-based paints, lead batteries, rubber products, and glass that find their way into the landfill. The mean concentration of Ba in soil and water did not exceed the limit set by US EPA (soil: 70–3000 mg kg^−1^, water: 2.0 mg L^−1^)^57^. Excess of Ba in soil and water may disturb the calcium metabolic process^58^. The mean concentration of Pb in both soil and water samples were found under the standard limit set by regulatory agencies like EU (soil: 50–300 mg kg^−1^)^46^, US EPA (soil: 200 mg kg^−1^, water: 0.05 mg L^−1^)^54^. Soil microbial mass containing carbon and nitrogen is considerably affected by the higher concentration of Pb present in it and deteriorate fertility by decreasing the rate of nutrient cycling^59^. In natural sources Pb is found in limited as compared to anthropogenic sources so its presence in environment is consider as indicator of pollution^10^. The mean concentration of Hg in both soil and water samples was found under the standard limit set by WHO (soil: 0.08 mg kg^−1^, water: 0.006 mg L^−1^) but the concentration of Hg in water exceeding the limit set by the US EPA (0.002 mg kg^−1^)^54^. Nematodes are one of the most species-rich metazoan phyla in soil ecosystems, live within the interstitial spaces of soils play a vital role in nutrient cycling in soil. These nematodes are affected by the high concentration of Hg. Exposure to human leading to defects in immune response, vital organs, nervous system and reproduction^60^.

Degradation of biodegradable material in landfill also plays an important role for leaching of THs and HMs into water and soil. Large amount of oxygen as oxidant present in the aquifer matrix that induces the reducing conditions and the existing state of these metals is disturbed in groundwater. Since the THs and HMs are covered by high quantities of activated and non-activated colloids, large and small dissolved particles, which resulted in the adsorption of these metals on the pore of the aquifer^9^. This is fact that Cr, As, Cd, and Pb have been found to contribute to large proportions of potential risk with respect to metal contamination in the soil due to human activities. They have been grouped in the priority metals that are important for public health and could damage the multiple organs and were categories as human carcinogens^45^.

### Toxic and heavy metals contamination assessment in soil

Geo-accumulation index (I_geo_), contamination factor (CF) and potential ecological risk index (PERI) were determined to assess pollution, evaluate the pattern of contamination and determine the potential risk due to exposure to ecological sensitivity, concentration and toxicity of TMs and HMs in soil. I_geo_ indicated (Table 5) that soil is uncontaminated to moderately contaminated with respect to all metals except the Cu and Pb which was categorized as uncontaminated. As with the distribution of I_geo_ the soil was considerable contaminated with respect to all metals except Cu (≈ 3.0), Cd (≈ 3.0), Pb (2.97) and was categorized as moderately contaminated. Data has shown (Table 5) that considerable ecological risk was measured with respect to soil contaminated with Cd and Hg while the low ecological risk was measured due to contribution from Cr, Cu, As and Pb. Furthermore, Cd and Hg were long known of their considerable environmental risk. In China, study on HMs composition in water for ecological risk assessment has shown that the contamination with these metals resulted in moderate to high risk. In the current study area, humans can become exposed to TMs and HMs when utilizing the contaminated soils for agricultural activities and accumulation of these in soils leads to increased phyto-accumulation in the crops grown.

**Table 5:** Geo-accumulation index (I_geo_), contamination factor (CF) and potential ecological risk index (PERI).

Index	TMs				HMs			
	Cr	Mn	Cu	As	Cd	Ba	Hg	Pb
I_geo_	1.14	1.22	1.00	1.19	1.02	1.06	1.19	0.98
CF	3.31	3.49	3.02	3.43	3.04	3.14	3.43	2.97
PERI	6.618	–	15.095	34.280	152.15	–	137.12	14.835

### Human health risk assessment

#### Average daily dose

ADD in mg^−1^ kg^−1^ day^−1^ of metals including Cr, Mn, Cu, As (TMs) and Cd, Ba, Hg, Pb (HMs) was calculated based on three exposure pathways of soil such as ADD_*ing*_, ADD_*inh*_, and ADD_*derm*_ while for water ADD_*ing*_ and ADD_*derm*_ were considered. ADD trend (Table 6) in both adults and children was found in order ADD_*ing*_ > ADD_*derm*_ > ADD_*inh*_ for soil while the same trend was observed for water in both groups (adults and children) such as ADD_*ing*_ > ADD_*derm*_. Among the ADD of soil, ADD_*ing*_ and ADD_*inh*_ for children had the maximum dose for all metals than adults while ADD_*derm*_ was higher in adults. In the case of water, the data showed more exposure of metals to children than adults. In conclusion, children are exposed to TMs and HMs more than adults.

**Table 6:** Results of ADD and HQ values from soil and water for adults and children.

Metal	ADD_*ing*_	ADD_*inh*_	ADD_*derm*_	HQ_*ing*_	HQ_*inh*_	HQ_*derm*_
**Soil**						
*Adults*						
Cr	1.112 × 10^−4^	1.635 × 10^−8^	4.767 × 10^−7^	3.706 × 10^−2^	5.716 × 10^−4^	7.945 × 10^−3^
Mn	2.179 × 10^−4^	3.204 × 10^−8^	9.342 × 10^−7^	1.556 × 10^−3^	2.288 × 10^−7^	5.190 × 10^−4^
Cu	5.199 × 10^−5^	7.645 × 10^−9^	2.228 × 10^−7^	1.299 × 10^−3^	1.901 × 10^−7^	1.856 × 10^−5^
As	1.014 × 10^−6^	1.492 × 10^−10^	1.305 × 10^−7^	3.380 × 10^−3^	4.973 × 10^−7^	1.060 × 10^−3^
Cd	4.931 × 10^−7^	7.252 × 10^−11^	2.114 × 10^−9^	4.931 × 10^−3^	7.252 × 10^−7^	2.114 × 10^−4^
Ba	7.617 × 10^−5^	1.120 × 10^−8^	3.265 × 10^−7^	1.088 × 10^−3^	7.832 × 10^−5^	6.663 × 10^−5^
Hg	1.691 × 10^−8^	2.486 × 10^−12^	7.248 × 10^−11^	5.636 × 10^−5^	8.286 × 10^−9^	3.451 × 10^−6^
Pb	2.205 × 10^−5^	3.242 × 10^−9^	9.452 × 10^−8^	6.300 × 10^−3^	9.975 × 10^−7^	1.800 × 10^−4^
*Children*						
Cr	1.038 × 10^−3^	7.632 × 10^−8^	4.177 × 10^−7^	3.460 × 10^−1^	2.668 × 10^−3^	6.961 × 10^−3^
Mn	2.034 × 10^−3^	1.495 × 10^−7^	8.185 × 10^−7^	1.452 × 10^−2^	1.067 × 10^−6^	4.547 × 10^−4^
Cu	4.852 × 10^−4^	3.568 × 10^−8^	1.952 × 10^−7^	1.213 × 10^−2^	8.875 × 10^−7^	1.626 × 10^−5^
As	9.468 × 10^−6^	6.962 × 10^−10^	1.143 × 10^−7^	3.156 × 10^−2^	2.320 × 10^−6^	9.292 × 10^−4^
Cd	4.603 × 10^−6^	3.384 × 10^−10^	1.852 × 10^−9^	4.603 × 10^−2^	3.384 × 10^−6^	1.852 × 10^−4^
Ba	7.109 × 10^−4^	5.227 × 10^−8^	2.861 × 10^−7^	1.015 × 10^−2^	3.655 × 10^−4^	5.838 × 10^−5^
Hg	1.758 × 10^−7^	1.160 × 10^−11^	6.350 × 10^−11^	5.860 × 10^−4^	3.866 × 10^−8^	3.023 × 10^−6^
Pb	2.058 × 10^−4^	1.513 × 10^−8^	8.281 × 10^−8^	5.880 × 10^−2^	4.655 × 10^−6^	1.577 × 10^−4^
**Water**						
*Adults*						
Cr	3.142 × 10^−11^	–	1.628 × 10^−11^	1.047 × 10^−8^	–	2.713 × 10^−7^
Mn	5.971 × 10^−10^		3.094 × 10^−9^	4.265 × 10^−9^	–	1.718 × 10^−6^
Cu	1.037 × 10^−9^	–	5.374 × 10^−10^	2.592 × 10^−8^	–	4.478 × 10^−8^
As	3.142 × 10^−10^	–	4.885 × 10^−9^	1.047 × 10^−6^	–	3.971 × 10^−5^
Cd	6.285 × 10^−11^	–	3.257 × 10^−11^	6.285 × 10^−7^	–	3.257 × 10^−6^
Ba	4.902 × 10^−9^	–	2.540 × 10^−9^	7.002 × 10^−8^	–	5.183 × 10^−7^
Hg	2.200 × 10^−10^	–	1.140 × 10^−10^	7.333 × 10^−7^	–	5.428 × 10^−6^
Pb	1.068 × 10^−9^	–	5.537 × 10^−10^	3.051 × 10^−7^	–	1.054 × 10^−6^
*Children*						
Cr	1.000 × 10^−10^	–	2.660 × 10^−11^	3.333 × 10^−8^	–	4.433 × 10^−7^
Mn	1.900 × 10^−9^	–	5.054 × 10^−10^	1.357 × 10^−8^	–	2.807 × 10^−7^
Cu	3.300 × 10^−9^	–	8.778 × 10^−10^	8.250 × 10^−8^	–	7.315 × 10^−8^
As	1.000 × 10^−9^	–	7.980 × 10^−9^	3.333 × 10^−6^	–	6.487 × 10^−5^
Cd	2.000 × 10^−10^	–	5.320 × 10^−11^	2.000 × 10^−6^	–	5.320 × 10^−6^
Ba	1.560 × 10^−8^	–	4.149 × 10^−9^	2.228 × 10^−7^	–	4.546 × 10^−7^
Hg	7.000 × 10^−10^	–	1.862 × 10^−10^	2.333 × 10^−6^	–	8.866 × 10^−6^
Pb	3.400 × 10^−9^	–	9.044 × 10^−10^	9.714 × 10^−7^	–	1.722 × 10^−6^

#### Total hazard quotient and index

HQ trend (Table 6) in both adults and children was found in order HQ_*ing*_ > HQ_*derm*_ > HQ_*inh*_ for soil for all metals except Ba which followed HQ_*ing*_ > HQ_*inh*_ > HQ_*derm*_. Whereas HQ trend in both adults and children was found in order HQ_*derm*_ > HQ_*ing*_ for water except the Cu in children was found in order of HQ_*ing*_ > HQ_*derm*_. HI values for an individual metal by all exposure pathways of soil and water in both adults and children was less than one which indicates no significant health risk. HI, values of Cr and Pb in children were 7 and 7.5 times higher than adults respectively. So, data from Table 7, demonstrated that the HI value for all metals in both soil and water is < 1 which indicated no significant health risk.

**Table 7:** Results of HI and LCR values from soil and water for adults and children.

Metal	HI	Interpretation	LCR	Interpretation
**Soil**				
*Adults*				
Cr	4.557 × 10^−2^	No significant risk	7.514 × 10^−5^	No carcinogenic risk
Mn	2.075 × 10^−3^	No significant risk	–	–
Cu	1.317 × 10^−3^	No significant risk	–	–
As	4.440 × 10^−3^	No significant risk	1.717 × 10^−6^	No carcinogenic risk
Cd	5.143 × 10^−3^	No significant risk	2.006 × 10^−7^	No carcinogenic risk
Ba	1.232 × 10^−3^	No significant risk	–	–
Hg	5.981 × 10^−5^	No significant risk	–	–
Pb	6.480 × 10^−3^	No significant risk	1.914 × 10^−7^	No carcinogenic risk
*Children*				
Cr	3.556 × 10^−1^	No significant risk	5.361 × 10^−4^	Carcinogenic risk
Mn	1.497 × 10^−2^	No significant risk	–	–
Cu	1.214 × 10^−2^	No significant risk	–	–
As	3.249 × 10^−2^	No significant risk	1.437 × 10^−5^	No carcinogenic risk
Cd	4.621 × 10^−2^	No significant risk	1.760 × 10^−6^	No carcinogenic risk
Ba	1.057 × 10^−2^	No significant risk	–	–
Hg	9.756 × 10^−4^	No significant risk	–	–
Pb	5.896 × 10^−2^	No significant risk	1.752 × 10^−6^	No carcinogenic risk
**Water**				
*Adults*				
Cr	2.817 × 10^−7^	No significant risk	6.831 × 10^−10^	No carcinogenic risk
Mn	1.722 × 10^−6^	No significant risk	–	–
Cu	7.070 × 10^−8^	No significant risk	–	–
As	4.075 × 10^−5^	No significant risk	7.798 × 10^−9^	No carcinogenic risk
Cd	3.885 × 10^−6^	No significant risk	2.289 × 10^−10^	No carcinogenic risk
Ba	5.883 × 10^−7^	No significant risk	–	–
Hg	6.161 × 10^−6^	No significant risk	–	–
Pb	1.359 × 10^−6^	No significant risk	3.233 × 10^−11^	No carcinogenic risk
*Children*				
Cr	4.776 × 10^−7^	No significant risk	1.140 × 10^−9^	No carcinogenic risk
Mn	2.942 × 10^−7^	No significant risk	–	–
Cu	1.556 × 10^−7^	No significant risk	–	–
As	6.820 × 10^−5^	No significant risk	1.347 × 10^−8^	No carcinogenic risk
Cd	7.320 × 10^−6^	No significant risk	4.111 × 10^−10^	No carcinogenic risk
Ba	6.774 × 10^−7^	No significant risk	–	–
Hg	1.119 × 10^−5^	No significant risk	–	–
Pb	2.693 × 10^−6^	No significant risk	6.688 × 10^−11^	No carcinogenic risk

#### Carcinogenic risk assessment

The carcinogenic slope factor was used to assess the carcinogenic risk from a lifetime exposure of TMs (Cr, Mn, Cu, As) and HMs (Cd, Ba, Hg, Pb) by different exposure pathways like ingestion, inhalation, and dermal. LCR < 10^−6^, LCR > 1 × 10^−4^, and LCR 1 × 10^−6^ to 1 × 10^−4^ indicates no carcinogenic risk, high risk of developing cancer, and signifies acceptable risk to humans respectively. LCR value for Cr by different exposure pathways of soil was 5.361 × 10^−4^ for children which are at the lower borderline of risk for cancer (Table 7). Therefore children are more at risk than adults in this study area where ingestion route is a major contributor to access LCR followed by dermal and inhalation pathways.

I_geo_, CF and PERI evaluated the pattern of contamination and determine the potential risk due to exposure to ecological sensitivity while the ADD, THQ, and CR coefficients were demonstrated to evaluate the human health risk, is the strength of present research. Whereas the remediation approach is to create a final solution that is protective of human health and the environment is limitation of this research work did on soil and water near landfill site.

## Conclusion

In this study, the concentration of toxic (Cr, Mn, Cu, As) and heavy metals (Cd, Ba, Hg, Pb) in soil and water samples near the landfill site of industrial waste in Sialkot, province of Punjab-Pakistan, was determined by ICP-OES. Toxic and heavy metals concentration in soil was found in following sequence Mn > Cr > Cu > As and Ba > Pb > Cd > Hg respectively whereas their concentration in water was found in following order Cu > Mn > As > Cr and Ba > Pb > Hg > Cd respectively. The mean concentration of all metals in both soil and water samples (n = 20, each) was found under the standard limit set by regulatory agencies like EU, US EPA, and WHO, except the Cr, this soil is not suitable for gardening because the Cr and Cd concentration found exceeds the limit set by US EPA. As with the distribution of I_geo_ the soil was considerable contaminated with respect to all metals except Cu (≈ 3.0), Cd (≈ 3.0), Pb (2.97) and was categorized as moderately contaminated. The results indicated that among the ADD of soil, ADD_*ing*_ and ADD_*inh*_ for children had the maximum dose for all metals than adults while ADD_*derm*_ was higher in adults and HI value for all metals in both soil and water is < 1 which indicated no significant health risk. In this study area, children are more at risk than adults where the ingestion route is a major contributor to access LCR followed by dermal and inhalation pathways. Based on the results of human health risk assessments, metals present in soil and water pose negligible non-carcinogenic and carcinogenic risks to the adults and children living in the area under study. But need for the attention and actions of the minimization and prevention of toxic and heavy metal contamination in soil and water by retardation of leachate movement via the appropriate design of new dumping sites with proper foundations. The present observations present useful information regarding the contamination of soil and water with toxic and heavy metals in an industrial area, also has much significance in the area where there are inappropriate practices in solid waste management.

Since these toxic and heavy metals can bio-accumulate, efforts should be made by Government agencies in alleviating their level in soil and water, and this research is useful in taking protective measures to save urban environment. Government agencies should consider new technologies with better capacities for removing these metals from landfill to prevent it discharge into soil and water. Construction of vertical engineered barriers (VEB), caps, and liners can be used to prevent the leaching of these metals into soil and water. So the human exposure to soil and water indicates the necessity of future interventions and policy responses.

## Supplementary Information

Supplementary Tables.
