# Supplementary material for: Toxic and heavy metals contamination assessment in soil and water to evaluate human health risk
Source: Sci Rep. 2021 Aug 20;11:17006. doi: 10.1038/s41598-021-94616-4 (PMC8379239; doi:10.1038/s41598-021-94616-4)
Supplement: Supplementary file 1 — Supplementary Tables. [file 41598_2021_94616_MOESM1_ESM.docx]

**Supplementary Data**

**Toxic and heavy metals contamination assessment in soil and water to evaluate human health risk**

Waqar Ahmad ^1^, Rima D. Alharthy ^2*^, Muhammad Zubair ^1*^, Mahmood Ahmed ^3*^, Abdul Hameed ^4^,

Sajjad Rafique ^1^

^1^ Department of Chemistry, University of Gujrat, Gujrat-Pakistan

^2^ Department of Chemistry, Science and Arts College, Rabigh Campus, King Abdulaziz University,

Jeddah 21577, Saudi Arabia

^3^ Renacon Pharma Limited, Lahore 54600, Pakistan

^4^ Department of Chemistry, University of Sahiwal, Sahiwal-Pakistan

**Running title:** Metals in soil and water

To whom correspondence should be addressed

Rima D. Alharthy, PhD, [iaaalharte@kau.edu.sa](mailto:iaaalharte@kau.edu.sa)

Muhammad Zubair, PhD, [muhammad.zubair@uog.edu.pk](mailto:muhammad.zubair@uog.edu.pk)

Mahmood Ahmed, PhD, [mahmoodresearchscholar@gmail.com](mailto:mahmoodresearchscholar@gmail.com)

**Table 1S.** Measured values of the metals in SRM 2709a and SRM 1640a

| Metal | SRM 2709a | SRM 1640a |
| --- | --- | --- |
| Cr | 131.24,134.11,132.35,135.55,131.54,132.55 | 40.58, 39.65, 38.65, 39.65, 38.11, 38.54 |
| Mn | 527.11, 528.53, 527.61, 529.82, 527.12, 527.17 | 39.54, 39.65, 38.96, 37.65, 39.11, 39.87 |
| Cu | 32.81, 33.25, 33.54, 33.68, 33.98, 33.65 | 85.44, 84.98, 85.46, 85.17, 85.97, 85.11 |
| As | 11.45, 10.98, 10.77, 11.25, 11.58, 11.48 | 7.65, 7.55, 8.11, 8.54, 8.08, 8.11 |
| Cd | 0.387, 0.398, 0.321, 0.361, 0.348, 0.354 | 3.58, 3.66, 3.88, 3.69, 3.88, 3.45 |
| Ba | 967.22, 968.58, 972.23, 975.32, 978.22, 978.11 | 151.25, 152.24, 151.26, 152.34, 152.98, 151.23 |
| Hg | 0.091, 0.084, 0.089, 0.085, 0.094, 0.091 | - |
| Pb | 17.58, 17.98, 17.58, 17.88, 17.96, 17.82 | 12.11, 12.07, 11.89, 11.85, 11.95, 11.45 |

**Table 2S.** Parameters of regression equation, detection limits and precision studies

| Element | Coefficient of determination (r^2^) | Intercept (b) | Slope (m) | LOD (μg /g) | LOQ (μg /g) | % RSD (soil/water) |
| --- | --- | --- | --- | --- | --- | --- |
| Cr | 0.9999 | 43.0 | 2854 | 0.154 | 0.515 | 1.20/2.09 |
| Mn | 0.9993 | 0.1525 | 0.1341 | 0.011 | 0.034 | 0.20/1.68 |
| Cu | 0.9992 | 0.1961 | 0.7526 | 0.019 | 0.065 | 1.03/0.42 |
| As | 0.9989 | 0.86 | 1906 | 0.083 | 0.276 | 2.44/3.37 |
| Cd | 0.9999 | 351.0 | 5939 | 0.262 | 0.874 | 6.40/4.06 |
| Ba | 0.9998 | 54.6 | 6043 | 0.036 | 0.120 | 0.46/0.54 |
| Hg | 0.9998 | 8.6 | 951.2 | 0.035 | 0.117 | 3.51/- |
| Pb | 0.9997 | 26.0 | 255.4 | 0.409 | 1.366 | 1.62/1.60 |

**Table 3S.** Precision studies for determination of metals in SRM 2709a and SRM 1640a

| Metal | SRM 2709a | SRM 1640a |
| --- | --- | --- |
| Cr | 131.24,134.11,132.35,135.55,131.54,132.55, 130.25, 131.24, 131.55, 130.98 | 40.58, 39.65, 38.65, 39.65, 38.11, 38.54, 40.11, 40.25, 39.88, 40.11 |
| Mn | 527.11, 528.53, 527.61, 529.82, 527.12, 527.17, 528.88, 529.11, 528.56, 529.77 | 39.54, 39.65, 38.96, 37.65, 39.11, 39.87, 39.54, 39.66, 39.44, 39.88 |
| Cu | 32.81, 33.25, 33.54, 33.68, 33.98, 33.65, 33.65, 33.54, 33.11, 33.78 | 85.44, 84.98, 85.46, 85.17, 85.97, 85.11, 85.44, 85.01, 85.88, 85.74 |
| As | 11.45, 10.98, 10.77, 11.25, 11.58, 11.48, 11.22, 11.45, 11.65, 11.45 | 7.65, 7.55, 8.11, 8.54, 8.08, 8.11, 8.09, 7.98, 8.01, 8.09 |
| Cd | 0.387, 0.398, 0.321, 0.361, 0.348, 0.354, 0.388, 0.358, 0.369, 0.387 | 3.58, 3.66, 3.88, 3.69, 3.88, 3.45, 3.65, 3.89, 3.56, 3.66 |
| Ba | 967.22, 968.58, 972.23, 975.32, 978.22, 978.11, 978.56, 978.44, 978.11, 978.88 | 151.25, 152.24, 151.26, 152.34, 152.98, 151.23, 150.98, 150.11, 151.44, 151.22 |
| Hg | 0.091, 0.084, 0.089, 0.085, 0.094, 0.091, 0.089, 0.088, 0.087, 0.092 | - |
| Pb | 17.58, 17.98, 17.58, 17.88, 17.96, 17.82, 18.01, 17.85, 17.45, 17.11 | 12.11, 12.07, 11.89, 11.85, 11.95, 11.45, 11.88, 11.98, 12.05, 12.06 |

|  | **Table 4S.** Soil samples results | | | | |  |  |  |
| --- | --- | --- | --- | --- | --- | --- | --- | --- |
|  | Metals | | | | | | | |
|  | Arsenic (As) | Barium (Ba) | Cadmium (Cd) | Chromium (Cr) | Copper (Cu) | Manganese (Mn) | Lead (Pb) | Mercury (Hg) |
|  | mg/kg | mg/kg | mg/kg | mg/kg | mg/kg | mg/kg | mg/kg | mg/kg |
| SLES1 | 2.1 | 125.0 | 1.0 | 165.0 | 80.0 | 320.0 | 35.0 | 0.025 |
| SLES2 | 2.0 | 125.0 | 0.8 | 160.0 | 80.0 | 300.0 | 22.0 | 0.025 |
| SLES3 | 2.0 | 120.0 | 0.8 | 120.0 | 66.0 | 298.0 | 22.0 | 0.020 |
| SLES4 | 1.0 | 110.0 | 0.7 | 120.0 | 40.0 | 270.0 | 20.0 | 0.020 |
| SLES5 | 1.0 | 105.0 | 0.6 | 114.0 | 40.0 | 270.0 | 17.0 | 0.020 |
| SLWS1 | 1.6 | 110.0 | 1.0 | 125.0 | 125.0 | 345.0 | 55.0 | 0.045 |
| SLWS2 | 1.5 | 105.0 | 0.9 | 105.0 | 120.0 | 320.0 | 35.0 | 0.042 |
| SLWS3 | 1.5 | 100.0 | 0.8 | 96.0 | 110.0 | 296.0 | 35.0 | 0.040 |
| SLWS4 | 1.5 | 96.0 | 0.7 | 80.0 | 110.0 | 280.0 | 22.0 | 0.040 |
| SLWS5 | 1.5 | 90.0 | 0.7 | 70.0 | 90.0 | 260.0 | 20.0 | 0.025 |
| SLNS1 | 2.0 | 150.0 | 0.6 | 110.0 | 85.0 | 350.0 | 55.0 | 0.035 |
| SLNS2 | 2.0 | 120.0 | 0.6 | 100.0 | 85.0 | 300.0 | 55.0 | 0.030 |
| SLNS3 | 1.9 | 103.5 | 0.5 | 98.0 | 85.0 | 300.0 | 50.0 | 0.030 |
| SLNS4 | 1.8 | 101.0 | 0.1 | 94.3 | 75.0 | 298.1 | 47.9 | 0.025 |
| SLNS5 | 1.6 | 95.0 | 0.1 | 65.0 | 70.0 | 265.0 | 40.0 | 0.025 |
| SLSS1 | 0.9 | 110.0 | 0.9 | 535.0 | 60.0 | 410.0 | 20.0 | 0.011 |
| SLSS2 | 0.8 | 106.0 | 0.9 | 480.0 | 45.0 | 350.0 | 20.0 | 0.010 |
| SLSS3 | 0.8 | 100.0 | 0.8 | 205.0 | 45.0 | 340.0 | 19.0 | 0.008 |
| SLSS4 | 0.8 | 98.0 | 0.8 | 170.0 | 35.0 | 325.0 | 18.0 | 0.006 |
| SLSS5 | 0.5 | 93.0 | 0.6 | 145.0 | 30.0 | 290.0 | 18.0 | 0.005 |

**Table 5S.** Water samples results

|  | Results | | | | | | | |
| --- | --- | --- | --- | --- | --- | --- | --- | --- |
|  | Arsenic (As) | Barium (Ba) | Cadmium (Cd) | Chromium (Cr) | Copper (Cu) | Manganese (Mn) | Lead (Pb) | Mercury (Hg) |
|  | mg/l | mg/l | mg/l | mg/l | mg/l | mg/l | mg/l | mg/l |
| SLEW1 | 0.011 | 0.160 | 0.001 | 0.002 | 0.030 | 0.025 | 0.040 | 0.010 |
| SLEW2 | 0.011 | 0.160 | 0.001 | 0.002 | 0.027 | 0.020 | 0.040 | 0.010 |
| SLEW3 | 0.010 | 0.140 | 0.001 | 0.001 | 0.027 | 0.020 | 0.040 | 0.007 |
| SLEW4 | 0.009 | 0.140 | 0.001 | 0.001 | 0.027 | 0.020 | 0.027 | 0.007 |
| SLEW5 | 0.008 | 0.140 | 0.001 | 0.001 | 0.020 | 0.020 | 0.020 | 0.007 |
| SLWW1 | 0.013 | 0.170 | 0.002 | 0.001 | 0.030 | 0.020 | 0.030 | 0.007 |
| SLWW2 | 0.011 | 0.145 | 0.002 | 0.001 | 0.025 | 0.020 | 0.030 | 0.006 |
| SLWW3 | 0.010 | 0.140 | 0.002 | 0.001 | 0.025 | 0.020 | 0.030 | 0.005 |
| SLWW4 | 0.010 | 0.120 | 0.002 | 0.001 | 0.025 | 0.015 | 0.030 | 0.005 |
| SLWW5 | 0.010 | 0.120 | 0.001 | 0.001 | 0.025 | 0.008 | 0.030 | 0.005 |
| SLNW1 | 0.011 | 0.160 | 0.002 | 0.002 | 0.035 | 0.010 | 0.045 | 0.008 |
| SLNW2 | 0.010 | 0.160 | 0.002 | 0.002 | 0.032 | 0.008 | 0.040 | 0.006 |
| SLNW3 | 0.010 | 0.160 | 0.002 | 0.001 | 0.032 | 0.006 | 0.035 | 0.006 |
| SLNW4 | 0.009 | 0.160 | 0.002 | 0.001 | 0.030 | 0.005 | 0.034 | 0.006 |
| SLNW5 | 0.009 | 0.158 | 0.001 | 0.001 | 0.030 | 0.001 | 0.030 | 0.004 |
| SLSW1 | 0.013 | 0.200 | 0.002 | 0.002 | 0.060 | 0.040 | 0.045 | 0.010 |
| SLSW2 | 0.013 | 0.196 | 0.002 | 0.002 | 0.057 | 0.038 | 0.043 | 0.009 |
| SLSW3 | 0.011 | 0.170 | 0.001 | 0.001 | 0.040 | 0.030 | 0.035 | 0.008 |
| SLSW4 | 0.010 | 0.170 | 0.001 | 0.001 | 0.040 | 0.030 | 0.035 | 0.008 |
| SLSW5 | 0.009 | 0.160 | 0.001 | 0.001 | 0.040 | 0.030 | 0.030 | 0.008 |
